# Principal component and factor analytic models in international sire evaluation

**DOI:** 10.1186/1297-9686-43-33

**Published:** 2011-09-23

**Authors:** Anna-Maria Tyrisevä, Karin Meyer, W Freddy Fikse, Vincent Ducrocq, Jette Jakobsen, Martin H Lidauer, Esa A Mäntysaari

**Affiliations:** 1 Biotechnology and Food Research, Biometrical Genetics, MTT Agrifood Research Finland,31600 Jokioinen, Finland; 2 Animal Genetics and Breeding Unit, University of New England, Armidale NSW 2351, Australia; 3 Department of Animal Breeding and Genetics, SLU, Box 7023, S-75007 Uppsala, Sweden; 4 UMR 1313 INRA, Génétique Animale et Biologie Intégrative, 78352 Jouy-en-Josas Cedex, France; 5 Interbull Centre, Department of Animal Breeding and Genetics, SLU, Box 7023, S-75007 Uppsala, Sweden

## Abstract

**Background:**

Interbull is a non-profit organization that provides internationally comparable breeding values for globalized dairy cattle breeding programmes. Due to different trait definitions and models for genetic evaluation between countries, each biological trait is treated as a different trait in each of the participating countries. This yields a genetic covariance matrix of dimension equal to the number of countries which typically involves high genetic correlations between countries. This gives rise to several problems such as over-parameterized models and increased sampling variances, if genetic (co)variance matrices are considered to be unstructured.

**Methods:**

Principal component (PC) and factor analytic (FA) models allow highly parsimonious representations of the (co)variance matrix compared to the standard multi-trait model and have, therefore, attracted considerable interest for their potential to ease the burden of the estimation process for multiple-trait across country evaluation (MACE). This study evaluated the utility of PC and FA models to estimate variance components and to predict breeding values for MACE for protein yield. This was tested using a dataset comprising Holstein bull evaluations obtained in 2007 from 25 countries.

**Results:**

In total, 19 principal components or nine factors were needed to explain the genetic variation in the test dataset. Estimates of the genetic parameters under the optimal fit were almost identical for the two approaches. Furthermore, the results were in a good agreement with those obtained from the full rank model and with those provided by Interbull. The estimation time was shortest for models fitting the optimal number of parameters and prolonged when under- or over-parameterized models were applied. Correlations between estimated breeding values (EBV) from the PC19 and PC25 were unity. With few exceptions, correlations between EBV obtained using FA and PC approaches under the optimal fit were ≥ 0.99. For both approaches, EBV correlations decreased when the optimal model and models fitting too few parameters were compared.

**Conclusions:**

Genetic parameters from the PC and FA approaches were very similar when the optimal number of principal components or factors was fitted. Over-fitting increased estimation time and standard errors of the estimates but did not affect the estimates of genetic correlations or the predictions of breeding values, whereas fitting too few parameters affected bull rankings in different countries.

## Background

Active international trade of semen and embryos of dairy cattle has created a need for global comparisons of genetic merit of sires. The International Bull Evaluation Service, Interbull, was established in 1983 to respond to this need. International breeding values of dairy bulls are currently estimated three times a year and they are expressed in the units of each member countries and are relative to each country's own base group of animals [1]. In order to accurately perform the evaluations, reliable genetic parameters, i.e., variance components and genetic correlations, are required.

Daughter groups in different countries are assumed to be genetically correlated but environmentally uncorrelated. Therefore, each biological trait under evaluation is treated as a different trait for each country participating in the international sire evaluation. Typically, some countries are very highly correlated. The multi-dimensionality and high genetic correlations create several problems such as over-parameterized models, increased sampling variances and an increased probability of parameters to be outside the boundaries of the parameter space, e.g. [2]. For restricted maximum likelihood (REML) estimation, these, in turn, complicate maximization of the likelihood and thus, exacerbate the time needed to estimate variance components. The number of countries participating in the international Holstein sire evaluation for protein yield in 2011 is 28. This requires estimation of a 28 × 28 (co)variance (VCV) matrix described by 406 parameters, if the genetic (co)variance matrix is considered to be unstructured. The current practice is to estimate this matrix by performing a number of separate analyses considering selected sub-sets of countries [3,4]. The resulting estimates are then combined to build up the complete VCV matrix. Typically, this results in a non-positive definite matrix and a "bending" procedure is applied to ensure that the overall matrix is valid [5].

Principal component (PC) and factor analytic (FA) models provide a highly parsimonious structure for the VCV matrix compared to the standard multi-trait model, e.g. [6,7] and they have, therefore, attracted considerable interest for their potential to ease the burden of the estimation process for multiple-trait across country evaluations (MACE) [8]. Both approaches decompose the genetic covariance matrix into pertaining matrices of eigenvalues and eigenvectors. Each eigenvector, i.e., PC, forms a linear combination of the traits, while the corresponding eigenvalue gives the variance explained. PC are independent of each other.

The aim of the PC method is to detect all necessary components explaining variation in multi-dimensional data without loosing any important information. The first PC explains the maximum amount of genetic variability in the data and each successive PC explains the maximum amount of the remaining variability. For highly correlated traits, only the leading PC have practical influence on genetic variation and PC with a negligible effect can be omitted without impairing accuracy of estimation. Furthermore, the parameter reduction results in a rank reduction and in a reduction of the dimension of the mixed model equations.

The FA method is related to the PC method but its approach is different. The traits studied are assumed to be linear combinations of a few latent variables, referred to as common factors. Any variance not explained by these is modelled separately, i.e. as trait-specific, by fitting corresponding specific factors. Due to the partitioning of variance into common and trait-specific variance, the number of factors needed to explain the variability in the data is normally notably smaller than the number of PC needed in the PC approach. Further, since the factors are assumed to be uncorrelated, substantial sparsity of the mixed model equation (MME) is gained compared to the standard unstructured multivariate analysis. However, the resulting (co)variance matrix is of full rank if all trait-specific variances are non-zero. Furthermore, factor axes can be rotated. Normally, this is done to ease their interpretation, but it also makes it possible to use the Cholesky parameterization that enhances the convergence rate of maximum likelihood estimation, e.g. [7,9].

Madsen et al. [10] were the first to suggest the use of reduced rank covariance matrices for MACE. Instead of using standard expectation-maximization algorithm for REML estimation of variance components for MACE, they studied the feasibility of exploiting an average-information (AI) algorithm that is known to be fast and effective. They developed an AI-REML algorithm, which evaluates for each round, whether or not the VCV matrix is positive definite. If a non-positive definite matrix is encountered, the original VCV matrix is decomposed and all eigenvalues less than the operational zero are replaced with a small positive number. Thus, their method is not a real reduced rank method in the sense that small or negative eigenvalues would have been removed. In turn, Leclerc et al. [11] studied both PC and FA approaches for a sub-set of well-linked base countries, performing dimension reduction for this sub-set and then estimating genetic correlations between the remaining and the base countries, keeping the genetic correlations among the base countries fixed. When applying the approach proposed by Leclerc et al. [11], special emphasis should be placed on selection of suitable base countries.

Mäntysaari [12] introduced a bottom-up PC approach that begins with a sub-set of countries and adds the remaining countries sequentially. By examining in each step whether or not the new country increases the rank of the genetic VCV matrix, the bottom-up approach only fits PC with non-negligible eigenvalues and thus avoids over-parameterized models. While this original study was performed with a simulated dataset, recent work has demonstrated the usefulness of this approach to estimate the variance components for MACE [13,14].

Typically, the conventional PC analysis is done after the complete VCV matrix has been estimated. Then, the matrix is decomposed and if possible, its dimension is reduced. Kirkpatrick and Meyer [15] suggested the direct estimation of the leading principal components (direct PC). However, this requires the appropriate rank to be known or to be estimated prior to the variance component analysis. Similarly, a VCV matrix imposing a FA structure can be estimated directly [6]. However, a too stringent parameter reduction should be avoided since selecting too low a rank can lead to biased estimates of genetic parameters [14,15]. This is, because the number of available parameters is no longer sufficient to describe the (co)variance structure of the model adequately, and part of the genetic variance will be re-partioned into the residual variance. Furthermore, with more than one matrix to be estimated, the reduced rank estimator can be inconsistent, i.e. pick up the wrong subset of PC [2]. The risk of this happening when relatively few PC are considered is high.

Both direct PC and FA approaches have been applied to beef cattle datasets and have demonstrated their potential to be used for large, multi-trait data sets, e.g. [16,17]. In addition, the direct PC approach proved to be an appealing method to estimate variance components for MACE in a recent study [14]. The objectives of this study are to evaluate the utility of the factor analytic approach for variance component estimation for MACE and to assess the impact of alternative parameterizations, both PC and FA, for practical prediction of breeding values with MACE.

## Methods

### Dataset

Protein yield data from the August 2007 Interbull Holstein evaluation were used. A sire model with sire-maternal grandsire pedigree of 106 003 individuals was employed. The dataset comprised 116 941 de-regressed breeding values from 25 countries [18]. The number of bulls per country varied from 145 to 23 380, with a mean of 4 678 (Table 1). Bulls were mainly used in one country; only 8% of the bulls (7 621) were used in more than one country and 0.3% of the bulls (286) in more than 10 countries. Common bulls were defined as bulls with daughters in both countries, without restrictions on the country of origin. The number of common bulls varied dramatically between countries, ranging from zero to 1 194. The number of common bulls was smallest between the French Red Holstein and other countries (min 0, max 73, mean 9) and largest between the USA and other countries (min 6, max 1 044, mean 410). For a more detailed description of the data, see [14].

**Table 1:** Variances ± standard errors for protein yield from the factor analysis fitting 9 factors and from the PC analysis fitting 19 PC

Country	Number of bulls	FA9			PC19
			
		Common	Country specific	Combined	
Canada	7 028	113.2 ± 2.2	8.5 ± 1.2	121.7 ± 1.9	121.3 ± 1.9
Germany	16 734	66.2 ± 1.3	6.0 ± 1.0	72.2 ± 0.8	72.2 ± 0.8
Denmark-Finland-Sweden	8 900	61.9 ± 1.1	4.1 ± 0.7	66.0 ± 1.0	66.0 ± 1.0
France	11 127	76.9 ± 1.5	7.4 ± 1.0	84.3 ± 1.2	84.4 ± 1.2
Italy	6 322	81.6 ± 1.8	4.5 ± 1.1	86.1 ± 1.4	86.1 ± 1.4
The Netherlands	9 696	73.4 ± 1.4	5.6 ± 0.9	79.0 ± 1.1	78.9 ± 1.1
USA	23 380	315.3 ± 4.6	15.9 ± 3.1	331.2 ± 3.4	331.1 ± 3.4
Switzerland	715	51.3 ± 2.0	0.0 ± 0.0	51.3 ± 2.0	51.9 ± 2.0
Great Britain	4 361	54.9 ± 1.1	0.0 ± 0.0	54.9 ± 1.1	54.9 ± 1.1
New-Zealand	4 253	21.6 ± 0.5	0.0 ± 0.0	21.6 ± 0.5	21.6 ± 0.5
Australia	4 950	20.6 ± 0.8	4.9 ± 0.6	25.5 ± 0.6	25.6 ± 0.6
Belgium	634	38.2 ± 2.1	4.7 ± 0.9	42.9 ± 2.0	43.0 ± 2.0
Ireland	1 260	19.5 ± 0.9	1.4 ± 0.5	20.9 ± 0.7	20.9 ± 0.7
Spain	1 499	50.0 ± 1.5	3.0 ± 0.5	53.0 ± 1.4	52.8 ± 1.4
Czech Republic	2 036	80.3 ± 2.8	0.0 ± 0.0	80.3 ± 2.8	80.1 ± 2.8
Slovenia	196	7.9 ± 0.8	0.0 ± 0.0	7.9 ± 0.8	8.1 ± 0.9
Estonia	472	55.6 ± 4.3	5.0 ± 2.8	60.6 ± 3.5	61.1 ± 3.5
Israel	773	76.7 ± 4.1	0.0 ± 0.0	76.7 ± 4.1	76.1 ± 4.1
Swiss Red Holstein	1 162	46.8 ± 2.1	2.2 ± 1.1	49.0 ± 1.9	48.0 ± 1.8
French Red Holstein	145	76.9 ± 8.6	0.0 ± 0.0	76.9 ± 8.6	80.4 ± 9.1
Hungary	1 898	64.4 ± 2.4	8.6 ± 1.3	73.0 ± 2.2	72.9 ± 2.2
Poland	5 071	31.4 ± 2.0	0.6 ± 1.8	32.0 ± 0.8	32.0 ± 0.8
South Africa	920	38.3 ± 2.3	0.0 ± 0.0	38.3 ± 2.3	37.8 ± 2.2
Japan	3 177	63.8 ± 1.6	0.0 ± 0.0	63.8 ± 1.6	64.3 ± 1.6
Latvia	232	15.7 ± 3.2	7.0 ± 2.8	22.7 ± 2.3	23.1 ± 2.3

### Random regression MACE sire model

The classical MACE model for the **i***^th ^*sire, denoted as:

(1) $$y_{i}=X_{i}b+Z_{i}u_{i}+\varepsilon_{i},$$

and the random regression (RR) MACE model, denoted as:

(2) $$y_{i}=X_{i}b+Z_{i}V\nu_{i}+\varepsilon_{i},$$

are equivalent but differently parameterized models. In both (1) and (2), **y***_i _*is a *n_i _*vector of national de-regressed breeding values for bull *i *and **b **is a vector of *t *country/trait effects. In (1), **u***_i _*is a vector of *t *different international breeding values for bull *i *and in (2), ***ν**_i _*is a vector of *t *regression coefficients for bull *i*. **X***_i _*and **Z***_i _*denote incidence matrices assigning observations to respective effects. Decomposing the *t *× *t *genetic co(variance) matrix of sire effects, Var(**u***_i_*) = **G **into **G **= **VDV***^T ^*with **D **a matrix of eigenvalues and **V **the corresponding matrix of eigenvectors, gives Var(***ν**_i_*) = **D**. In (1) and (2), *ε_i _*is a *n_i _*vector of residuals with Var(*ε_i_*) = diag(*g_jj_λ_j_*/*EDC_ij_*), where *g_jj _*is the sire variance, $\lambda_{j}=\left(4-h_{j}^{2}\right)∕h_{j}^{2}$ with $h_{j}^{2}$ the heritability of country *j *and *EDC_ij _*the effective daughter contribution of bull *i *in country *j*. In (2), the breeding values of bull *i *have to be back-transformed to get **u***_i _*= **V*ν***_*i*_. For the estimation of variance components, we did not group animals with unknown parentage into genetic groups, but for prediction of the breeding values, genetic groups were used.

### PC approach

The RR MACE model facilitates parameter reduction, when **G **has eigenvalues close to zero. Then, the principal components with the smallest eigenvalues can be omitted without impairing the accuracy of estimation. In that case, **G **can be described as $G_{\text{1}}=V_{\text{1}}D_{\text{1}}V_{1}^{T}$, where **D**_1 _is *r *× *r *and contains the *r *leading eigenvalues and **V**_1 _is the *t *× *r *matrix of the *r *corresponding eigenvectors, with *r *<*t*. Now, the *r *random regression coefficients, $\nu_{i}^{*}$, are predicted for each bull and the breeding values can be back-transformed: $u_{i}≅V_{1}D_{1}\nu_{i}^{*}$.

### FA approach

For the FA approach, **u***_i _*is divided into vectors of common factors, ***δ***_*i*_, with Var(***δ***_*i*_) = **I**, and country specific effects, ***τ***_*i*_, with $\text{Var}(\tau_{i})=F=\text{diag\{}\sigma_{\tau_{ij}}^{2}\}$. This gives **u***_i _*= **L*****δ**_i _*+ ***τ***_*i*_, with **L **denoting the matrix of factor loadings [19]. The FA representation of the MACE model is expressed as:

(3) $$y_{i}=X_{i}b+Z_{i}\left(L\delta_{i}+\tau_{i}\right)+\varepsilon_{i}$$

The FA approach models **G **as the sum of two terms: the common (co)variances and the trait-specific variances, i.e., **G **= **LL***^T ^*+ **F**. The number of parameters can not exceed *t*(*t *+ 1)/2, thus *r *<*t *factors explain the common covariances, e.g. [7]. If all country-specific variances are non-zero, the resulting model will not be of reduced rank, but is described very parsimoniously with *p *= *t *+ *rt *- *r*(*r *- 1)/2 [7].

### Models

Contrary to the current practice of data sub-setting for MACE variance component analysis for protein yield in Holstein [3], all the data in this study was included in a single VCV analysis for each model investigated. For the PC approach, estimates of **G **from several fits were obtained from a previous study [14]. The appropriate fit was chosen by performing several analyses encompassing a first, informed guess of the correct rank, which was obtained by decomposing the (co)variance matrix provided by Interbull and by studying the magnitude of the eigenvalues. Next, we examined Akaike's information criterion (AIC), log L and behaviour of the PC from analyses using successive numbers of PC to determine the appropriate rank. For the model with an optimal fit, AIC should reach its minimum value and the increase of the Log Likelihood beyond the optimal fit is expected to be marginal. Furthermore, the magnitude of the leading PC and the sum of the eigenvalues should be stabilized, i.e. not change value as the number of PC fitted is increased. If this were not the case, it would be an indication that there was still notable re-partioning of the genetic variance into the residual variance, i.e. that too few PC had been fitted [2,16,17]. For the direct PC approach, rank 19 (PC19) was selected as best [13,14]. For comparison, analyses were also carried out using too low a rank (PC15) and full rank (PC25).

For the FA approach, successive analyses fitting from seven to 12 factors were carried out and the best model was chosen following the same principles as for the PC approach. A model fitting nine factors (FA9) was chosen as best and results from the model fitting too few factors (FA7) are presented for comparison. In addition, $\sqrt{\Delta r}$ values, defined as the square root of the average squared deviation of the estimated genetic correlations [17,14], were calculated to indicate the differences in the estimates of the genetic correlations between each tested fit and the reference model (PC19) for comparison.

(4) $$\sqrt{\Delta r}=\sqrt{2\overset{t}{\underset{i=1}{\sum }}\overset{t}{\underset{j=i+1}{\sum }}\frac{{\left(r_{ij,m}-r_{ij,19}\right)}^{2}}{t\times \left(t-1\right)}},$$

where *t *is the number of traits, *r*_*ij*,*m *_is the estimated genetic correlation between traits *i *and *j *from an analysis fitting *m *factors.

Estimated factors of the FA9 model were rotated to ease their interpretation. The purpose of rotating factors is to load variables as unambiguously as possible to the factors. In that case, each factor has only a small group of variables with strong loadings. Rotations can be classified into two groups: orthogonal and oblique rotations. In orthogonal rotation, factors do not correlate with each other:

(5) $$G=LTT^{T}L^{T}+F=LL^{T}+F,$$

where **T **is an orthogonal transformation matrix. In oblique rotation, axes do not remain perpendicular. In this case, the rotation uses a general non-singular transformation matrix instead of the orthogonal transformation matrix [19]. By allowing factors to correlate with each other, it is easier to cluster variables and simplify their interpretation. In this study, we performed the oblique promax rotation. Calculations were carried out using the R Stats package [20]. After rotation, the matrix was sorted and the smallest loadings (with a cut-off of 0.2) were hidden to further ease interpretation.

The number of parameters was 271, 305, 326, 180 and 215 for PC15, PC19, PC25, FA7 and FA9, respectively. Variance components were estimated by restricted maximum likelihood, using an average information algorithm as implemented in WOMBAT [21]. The genetic correlations obtained from the Interbull test run preceding August 2007 evaluation were used for comparison.

### Analysis of estimated breeding values

Consequences of applying the obtained variance components for the more parsimonious PC and FA models for the practical prediction of breeding values with MACE were studied by monitoring the correlations between estimated breeding values (EBV) from the different PC and FA models. For this, EBV were predicted under the following models: PC25, which is equal to the classical MACE model, PC and FA models with the optimal fit (PC19 and FA9) and PC and FA models with too low a fit (PC15 and FA7). Furthermore, correlations between EBV from PC15 and PC19, from FA7 and FA9, and from PC19 and FA9 for each country were considered for four subgroups: A) bulls used only in their own country, B) bulls used in their own country and abroad, C) bulls used only abroad, and D) imported bulls. Breeding values were obtained using a preconditioned conjugated gradient iteration on data algorithm as implemented in MiX99 [22].

## Results and Discussion

### Selection of the FA model

The information used for model selection for the FA approach is collected in Table 2. Based on AIC, fitting 9 factors was best, although the difference between FA9 and FA10 was very small. Mean values of the genetic correlations from the different fits were practically identical, although there were some differences in the distributions of the estimates from the different fits. Interestingly, based on the $\sqrt{\Delta r}$ values, genetic correlations from FA12 were closest to the estimates from the direct PC analysis under the optimal rank 19, but not to the genetic correlations from the optimal fit (FA9).

**Table 2:** Characteristics for the analyses fitting from seven to 12 factors

	Fit 7	Fit 8	Fit 9	Fit 10	Fit 11	Fit 12
-1/2 AIC^a^	-11	-17	0	-1	-8	-12
Log L^b^	-79	-67	-33	-18	-10	0
No of parameters	180	198	215	231	246	260
Sum of eigenvalues^c^	1541	1585	1602	1608	1619	1631
E1^d^	85.9	83.1	82.6	82.3	82.0	81.3
E2	4.7	4.9	4.8	4.7	4.3	4.4
E3	3.4	4.5	3.8	3.8	3.9	3.8
E4	2.4	2.6	2.9	2.9	2.7	2.8
E5	1.5	1.7	1.9	1.9	1.9	1.9
E6	1.2	1.4	1.5	1.5	1.7	1.6
E7	0.9	1.1	1.1	1.1	1.1	1.2
E8		0.7	0.8	0.9	0.8	0.9
E9			0.7	0.7	0.7	0.7
E10				0.4	0.6	0.6
E11					0.3	0.4
E12						0.3
*r_g_*, min^e^	0.16	0.05	0.13	0.12	0.07	0.06
*r_g_*, max^e^	0.93	0.94	0.94	0.94	0.94	0.94
*r_g_*, mean^e^	0.69	0.69	0.69	0.69	0.69	0.68
^${\sqrt{\Delta r}}^{\text{f}}$^	0.039	0.038	0.023	0.022	0.019	0.017

^a ^Akaike's information criterion, expressed as deviation from highest value

^b ^Maximum Log Likelihood, expressed as deviation from highest value

^c ^Derived from the variance due to common factors

^d ^Eigenvalues 1 to 12 of **LL***^T^*, expressed as proportion (in %) of total

^e ^Genetic correlations: minimum, maximum and mean values

^f ^Square root of the average squared deviation of the genetic correlations. The estimates obtained under the direct PC rank 19 model were used as the estimates of comparison.

Inspection of the sum of the eigenvalues derived from the variance due to common factors (Table 2) and the country-specific variances from the different fits revealed that some re-partioning of the genetic variance occurred with decreasing fit. Part of the variance due to common factors was moved into the country-specific variance. As a consequence, the number of countries with zero country-specific variance decreased from 13 (fit 12) to five (fit 7) and the sum of the country-specific variances increased by 57%. Except for the first eigenvalue, the distribution of the variance due to common factors for the individual eigenvalues remained, however, quite constant between the fits.

**Figure 1:**
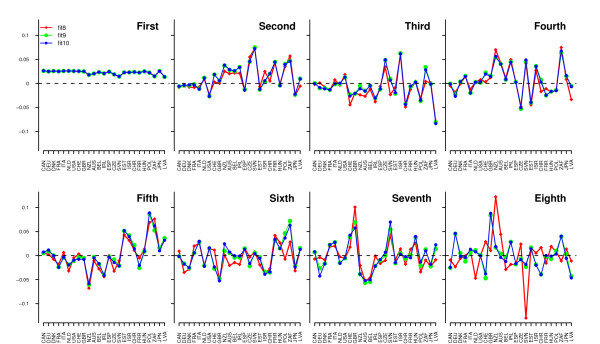
**First eight standardized eigenvectors from factor analysis under fits 8, 9 and 10**. Country codes: Canada (CAN), Germany (DEU), Denmark-Finland-Sweden (DFS), France (FRA), Italy (ITA), The Netherlands (NLD), United States of America (USA), Switzerland (CHE), Great Britain (GBR), New Zealand (NZL), Australia (AUS), Belgia (BEL), Ireland (IRL), Spain (ESP), Czech Republic (CZE), Slovenia (SVN), Estonia (EST), Israel (ISR), Swiss Red Holstein (CHR), French Red Holstein (FRR), Hungary (HUN), Poland (POL), South Africa (ZAF), Japan (JPN), Latvia (LVA).

The first eight eigenvectors from the optimal fit (FA9) and the two bracketing fits, i.e. FA8 and FA10, are shown in Figure 1. As might be expected from the almost identical AIC values for FA9 and FA10, all their eigenvectors were virtually identical. However, eigenvectors from analyses fitting eight factors started to deviate from those fitting nine and 10 factors from the second eigenvector onwards, with a substantial deviation for the eighth eigenvector. The pattern of the eigenvectors from FA7 deviated even more from those for the optimal fit (results not shown), indicating that fitting too few factors was associated with inaccurate estimation of the directions of the PC. In addition, overfitting PC had hardly any influence on estimation of the directions. This was not only the case for FA10, but also for FA11 and FA12 (results not shown). Results also indicated that the last eigenvectors of all tested fits were inaccurately estimated since their pattern deviated notably from the patterns of the eigenvectors of the models fitting more PC. Based on the simulation studies by Kirkpatrick and Meyer [15] and Meyer [16], inaccurate estimation of the last eigenvectors was caused by large sampling variances. However, this is of minor practical importance since the magnitude of the last eigenvalues is negligible compared to that of the leading eigenvalues, i.e. the last principal components contribute little to the estimate of the genetic covariance matrix (Table 2).

The matrix of rotated factor loadings is given in Table 3. Even with the rotation, their interpretation was not easy. In most cases, the possible interpretation seemed to be connected with the active trade of bulls between some countries and thus, with the strong genetic links created between them, see, e.g. [23]. Israel, South Africa and Japan import bulls predominantly from the USA, whereas the French Red population has only few links with the USA (factor 3). Furthermore, the highest proportion of imported bulls in Estonia, Poland and Latvia comes from Germany (factor 5). USA, France, Italy, Spain and Hungary have, in turn, strong links among others, mainly due to the trade of bulls from USA (factor 7), whereas the Netherlands is a popular trading partner with countries like Germany, Denmark, Finland, Sweden, Belgium and Ireland (factor 8). New-Zealand, Australia and Ireland were positively weighted countries in factor 9. The common feature for these is that they all are grazing countries.

**Table 3:** Rotated matrix of factor loadings from the FA9 analysis

	Factors								
	
Country	F1	F2	F3	F4	F5	F6	F7	F8	F9
Great Britain	-0.92								
Czech Republic		0.93							
Israel		0.24	0.89	0.26					
South Africa			0.23	0.95					
Estonia					0.61				
Poland				0.32	0.55				
Switzerland						-0.60			
Swiss Red Holstein						-0.55			
USA						0.26	-0.85		
Germany	-0.20				0.28	0.22		-0.54	
The Netherlands								-0.55	
New-Zealand								0.20	0.82
Canada						-0.29			-0.20
Denmark-Finland-Sweden								-0.28	
France						-0.27	-0.20		
Italy							-0.34		
Australia									0.43
Belgium								-0.40	
Ireland								-0.23	0.21
Spain							-0.20		
Slovenia				0.22	-0.20				
French Red Holstein		0.32	-0.39						
Hungary							-0.20		
Japan			0.25						
Latvia					0.23	-0.37			

### Variances and genetic correlations

Estimates of genetic variances from FA9, PC19 and PC25 were almost identical (FA9 and PC19 in Table 1), except for some differences between approaches for French Red Holstein (PC19: 80.4 ± 9.06, PC25: 80.6 ± 9.16, FA9: 76.9 ± 8.60). The differences in estimates and their high standard errors can be attributed to the low number of the bulls (145) in this population (Table 1). For FA9, there was substantial variation in the amount of the country specific variance. On average, the proportion of the total genetic variance attributed to country specific effects was 5%, with the highest proportions for Australia (19%) and Latvia (31%). Under the optimal fit (FA9), in nine of the 25 countries/populations the genetic variance was totally explained by the common variance. These countries/populations were Switzerland, Great Britain, New Zealand, Czech Republic, Slovenia, Israel, French Red Holstein, South Africa and Japan.

**Figure 2:**
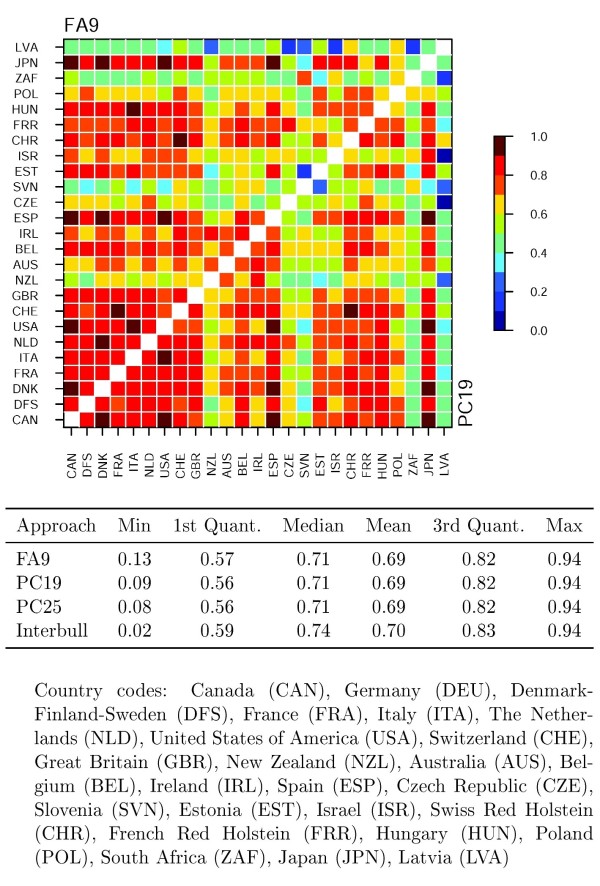
**Estimates of genetic correlations for protein yield from FA9 and PC19 analyses**. Summary statistics for Interbull and the PC full fit estimates are presented for comparison. Quant. Refers to quantile.

As shown in Figure 2, estimates of the genetic correlations for the FA and the direct PC approaches under the optimal fit were in good accordance. Further, Interbull and the direct PC full rank estimates presented for comparison (Figure 2), as well as the estimates from the bottom-up PC approach [13,14], were consistent with these estimates. Standard errors of the estimates from the direct PC full rank model were larger compared to those obtained under the optimal fit PC and FA models. Thus, parameter reduction using factor analytic, direct and bottom-up PC models worked well for variance component estimation for MACE. Furthermore, compared to the analyses using under- or over-parameterized models, optimal fit resulted also in the shortest running times: FA7 14.5 days, FA9 3.5 days, FA11 31.5 days, and PC15 21.5 days, PC19 9 days, PC25 16.5 days.

### Consequences of the PC and FA models for estimated breeding values

Correlations between EBV from the PC and FA approaches are in Tables 4 and 5. Results from the complete data are in Table 4 and results from the subsets of data in Table 5. EBV from PC19 and PC25 were identical. This was expected, given that the eigenvalues from 21 to 25 under the full rank model were zero [14]. The result shows that applying a PC model with the optimal fit has no practical consequences on ranking of the bulls. EBV correlations between PC15 and PC19 were lower than those between PC19 and PC25, demonstrating that the use of too low a rank affected the estimates. Results also indicated that the prediction of the EBV might be more sensitive when using too low a fit under the direct PC approach than under the factor analytic approach (Tables 4 and 5).

**Table 4:** Correlations between EBV in the complete data: analyses with optimal and too low a fit within approaches, and analyses with optimal fits between approaches

Country	PC15 PC19	FA7 FA9	FA9 PC19
Canada	0.999	1.000	1.000
Germany	1.000	1.000	1.000
Denmark-Finland-Sweden	1.000	1.000	1.000
France	1.000	1.000	1.000
Italy	1.000	1.000	1.000
The Netherlands	1.000	1.000	1.000
USA	1.000	1.000	1.000
Switzerland	0.999	1.000	1.000
Great Britain	1.000	1.000	1.000
New-Zealand	0.995	0.997	0.999
Australia	1.000	1.000	1.000
Belgium	0.997	1.000	1.000
Ireland	0.997	0.999	0.999
Spain	1.000	1.000	1.000
Czech Republic	0.993	0.997	0.999
Slovenia	0.994	0.993	0.979
Estonia	0.995	1.000	0.996
Israel	0.985	0.985	0.993
Swiss Red Holstein	0.999	1.000	0.999
French Red Holstein	0.999	0.988	0.988
Hungary	0.999	1.000	1.000
Poland	0.999	1.000	0.999
South Africa	0.992	0.995	0.998
Japan	0.999	0.999	1.000
Latvia	0.982	0.993	0.977

**Table 5:** Correlations between EBV in four subgroups: analyses with optimal and too low a fit within approaches, and analyses with optimal fits between approaches

	Subgroup B^a^			Subgroup C^b^			Subgroup D^c^		
			
Country	PC15 PC19	FA7 FA9	FA9 PC19	PC15 PC19	FA7 FA9	FA9 PC19	PC15 PC19	FA7 FA9	FA9 PC19
Canada	1.000	1.000	1.000	0.999	1.000	1.000	1.000	1.000	1.000
Germany	1.000	1.000	1.000	1.000	1.000	1.000	1.000	1.000	1.000
Denmark-Finland-Sweden	1.000	1.000	1.000	1.000	1.000	1.000	1.000	1.000	1.000
France	1.000	1.000	1.000	1.000	1.000	1.000	1.000	1.000	1.000
Italy	1.000	1.000	1.000	1.000	1.000	1.000	1.000	1.000	1.000
The Netherlands	1.000	1.000	1.000	1.000	1.000	1.000	1.000	1.000	1.000
USA	1.000	1.000	1.000	1.000	1.000	1.000	1.000	1.000	1.000
Switzerland	0.999	1.000	1.000	0.999	1.000	1.000	0.998	1.000	1.000
Great Britain	1.000	1.000	1.000	1.000	1.000	1.000	1.000	1.000	1.000
New-Zealand	0.998	0.999	1.000	0.994	0.997	0.999	0.998	0.999	1.000
Australia	1.000	1.000	1.000	1.000	1.000	1.000	1.000	1.000	1.000
Belgium	0.999	1.000	1.000	0.997	1.000	1.000	0.999	1.000	1.000
Ireland	0.999	1.000	1.000	0.997	0.999	0.999	1.000	1.000	1.000
Spain	1.000	1.000	1.000	1.000	1.000	1.000	1.000	1.000	1.000
Czech Republic	0.998	0.999	1.000	0.993	0.997	0.999	0.998	0.999	1.000
Slovenia	0.998	0.998	0.994	0.994	0.993	0.979	0.999	0.998	0.996
Estonia	0.999	1.000	1.000	0.995	1.000	0.996	0.999	1.000	1.000
Israel	0.995	0.990	0.996	0.985	0.985	0.993	0.995	0.990	0.996
Swiss Red Holstein	1.000	1.000	0.999	0.999	1.000	0.999	1.000	1.000	1.000
French Red Holstein	1.000	1.000	1.000	0.999	0.988	0.988	1.000	1.000	1.000
Hungary	0.998	1.000	1.000	0.999	1.000	1.000	0.999	1.000	1.000
Poland	0.999	1.000	1.000	0.998	0.999	0.999	0.999	1.000	1.000
South Africa	0.997	0.998	0.999	0.992	0.995	0.998	0.997	0.998	0.999
Japan	0.999	1.000	1.000	0.999	0.999	1.000	1.000	1.000	1.000
Latvia	0.996	0.999	0.996	0.982	0.993	0.977	0.998	0.999	0.998

^a ^Subgroup B: bulls have been used in their own country and abroad

^b ^Subgroup C: bulls have been used only abroad

^c ^Subgroup D: imported bulls

In most cases, EBV correlations from the FA and PC approaches under the optimal fit were unity or close to unity (Tables 4 and 5). EBV correlations were less than 0.99 only for Slovenia, the French Red Holstein population and Latvia in the complete dataset (Table 4). These were the countries/populations with the lowest number of records and weak ties with the other countries. The mean number of common bulls between French Red Holstein and the other countries was as low as 9, and those for Latvia and Slovenia were 29 and 32, respectively. Thus, the results indicate that the use of the FA approach under the optimal fit has no practical consequenses on the ranking of bulls.

All EBV correlations were unity in the subgroup A (bulls used only in their own country). In all studied subgroups, EBV correlations from the FA9 and PC19 were unity or close to unity, except in subgroup C (bulls used only abroad) for Slovenia, French Red Holstein and Latvia (< 0.99, Table 5). Correlations between EBV from PC15 and PC19 and from FA7 and FA9 tended to be lower than those between FA9 and PC19, but they were still very high. The only exceptions with correlations of 0.99 or greater occurred in subgroup C for Israel, French Red Holstein and Latvia. It was expected that subgroup C would be the most challenging group to analyse since the EBV were predicted based on correlated information only. Results agreed well with a previous study, in which the FA and PC approaches under the reduced rank RR MACE models were applied, but in which the variance components used for the predictions were provided by Interbull [24].

Using the PC19 model reduced the number of equations in the mixed model by 24% compared to PC25. For simplicity, the FA model was implemented as a standard multivariate model using **G **with a FA structure, instead of an extended FA model discussed by Thompson et al. [6]. Due to this, model FA9 provided no advantage of using more sparse coefficient matrix in the MME. Times required for solving mixed model equations ranged from 5 min (PC19) to 7 min (FA9). Thus, differences in the computing times were of no practical significance.

## Conclusions

The random regression representation of MACE facilitates exploitation of principal component or factor analytic approaches for variance component estimation and prediction of breeding values for international sire evaluations. Both PC and FA allow a reduction of the number of parameters to be estimated, and both methods benefit from the more parsimonious variance structure. Genetic parameters from different approaches were very similar when the optimal number of PC/factors was fitted. Computing time for estimation of variance components was shortest under the optimal fit. Overfitting increased the standard errors of the estimates, but had no visible impact on the estimates or on prediction of the breeding values. Fitting too low a number of parameters affected, in turn, bull rankings in different countries.

## Competing interests

The authors declare that they have no competing interests.

## Authors' contributions

AMT performed the statistical analyses and wrote the first draft of the manuscript. KM modified the WOMBAT software for the needs of this study. JJ and WFF provided the datasets. EAM, MHL, KM, VD, WFF and JJ supervised the study and contributed to writing the manuscript. All authors have read and approved the final manuscript.
